# A MRI Denoising Method Based on 3D Nonlocal Means and Multidimensional PCA

**DOI:** 10.1155/2015/232389

**Published:** 2015-10-12

**Authors:** Liu Chang, Gao ChaoBang, Yu Xi

**Affiliations:** ^1^School of Computer Science, Chengdu University, Chengdu 610106, China; ^2^Key Laboratory of Pattern Recognition and Intelligent Information Processing of Sichuan, Chengdu, China

## Abstract

Recently nonlocal means (NLM) and its variants have been applied in the various scientific fields extensively due to its simplicity and desirable property to conserve the neighborhood information. The two-stage MRI denoising algorithm proposed in this paper is based on 3D optimized blockwise version of NLM and multidimensional PCA (MPCA). The proposed algorithm takes full use of the block representation advantageous of NLM3D to restore the noisy slice from different neighboring slices and employs MPCA as a postprocessing step to remove noise further while preserving the structural information of 3D MRI. The experiments have demonstrated that the proposed method has achieved better visual results and evaluation criteria than 3D-ADF, NLM3D, and OMNLM_LAPCA.

## 1. Introduction

As a significant imaging technique, magnetic resonance imaging (MRI) provides very important information to research the tissues and organs in the human body with noninvasive style. However, MRI is affected by several artifacts and noise sources. One of them is the random fluctuation of the MRI signal which is mainly due to thermal noise. Such noise seriously degrades the acquisition of any quantitative measurements from the data. Consequently, the denoising techniques are required to improve the quality of MRI.

Generally speaking, MRI denoising techniques can be classified as either filtering, transform, or statistical approach [1]. Filtering methods remove noise with linear or nonlinear filters [2–5]. Transform methods employ some kinds of transformation to denoising MRI including wavelet transform [6] and curvelet transform [7]. Statistical methods estimate the noise with maximum likelihood [8], linear minimum mean square error (LMMSE) [9], Markov random process, and empirical Bayes [10]. In particular, nonlocal means (NLM) filter [11] has been used to denoise MRI image, achieving notable results [12–14]. NLM exploits the redundancy of the neighborhood pixel to remove the noise. The restored pixel is considered as the weighted average of the intensities of all pixels within the neighborhood area. Since MRI image has multichannel nature, NLM has been modified to denoise MRI data where the similarity measure can be considered to combine the relative information between different slices [15]. Nevertheless, the high computational burden has restricted its application for 3D MRI data. Therefore, [12] has proposed an optimized blockwise NLM filter for 3D MRI.

Due to its ability to perform decorrelation, PCA has also been used in image denoising. However, PCA requires that the number of images be bigger than the number of significant components of the image. The drawback has limited the application of PCA in the field of image denoising. The paper [16] has developed a two-stage approach to improve the quality of MRI data. After denoising with optimized* multicomponent* nonlocal mean (OMNLM), the local PCA is conducted over small local windows instead of the whole image to overcome the drawback. Nevertheless, PCA on overlapping windows will reduce the computational efficiency. Furthermore, the vectorization will make the structural information of image lost.

Actually, MRI is naturally a 3D image, which can be considered as tensor data on multidimensional space. From the aspect of super-resolution reconstruction, the noise image can be considered as the degraded version of the original image. Therefore, this paper proposed a multidimensional structure preserving MRI denoising algorithm. The algorithm consists of two stages. On the first stage, the 3D variant of the nonlocal means technique is employed to reduce the noise, which takes full advantage of the neighbor information between different 3D MRI slices and has the capability of exploiting the underlying structure in the multidimensional image. On the second stage, for the result image obtained from the first stage, multidimensional principal component analysis is performed to suppress the remaining noise, which avoids the vectorization to preserve the neighborhood information for MRI image and is helpful to improve the computation cost. According to the experiments on 3D MRI image, the proposed algorithm is superior to restore the original image from noise compared with other state-of-the-art methods.

## 2. Material and Methods

### 2.1. The 3D Variant of Nonlocal Mean

For image denoising problem, the noisy data *Y* is defined by the original noise free data *X* with some noise *N*:

(1) $$\begin{array}{c} Y=X+N. \end{array}$$

The classical NLM technology has believed that the intensity ${\hat{x}}_{i}$ of the point *i* can be restored from the weighted average of all the point intensities from the noisy image *X* based on the redundant representations of image [13]:

(2) x^i=∑j∈Ωwi,jxjs.t. 0≤wi,j≤1,  ∑j∈Ωwi,j=1,

where *w*(*i*, *j*) is the weight assigned to point *j* in the restoration of point *i* and *Ω* is the search area centered at the current point *i*. According to (2), this method has the capability to reconstruct the voxel from all similar voxels in the restricted neighbor volume *Ω*. Consequently, redundant information from the same MRI image and different slices can be used to reconstruct the current voxel efficiently.

The key issue of NLM is the computation of *w*(*i*, *j*), which represents the similarity of neighborhood points. Generally speaking, within the search area *Ω*, the weight *w*(*i*, *j*) is related to the distance *d*(*N*_*i*_, *N*_*j*_), with *N*_*i*_ and *N*_*j*_ being neighborhoods around *i* and *j* as follows [13]:

(3) $$\begin{array}{c} w\left(\;i,j\;\right)=\frac{1}{Z\left(i\right)}e^{-d\left(N_{i},N_{j}\right)/h^{2}}, \end{array}$$

where *Z*(*i*) is a normalization constant with *Z*(*i*) = ∑_*Ω*_ *w*(*i*, *j*) and *h* is a filtering parameter.

However, the basic NLM has a great influence for computational efficiency, especially for 3D MR images. Consequently, [12] has implemented the 3D blockwise version of NLM (NLM3D), which divides the volume into overlapping blocks and treats each block as a point to perform NLM-like restoration. For NLM3D, the *N*_*i*_ and *N*_*j*_ in (3) are 3D patches around points *i* and *j*, and *d*(*N*_*i*_, *N*_*j*_) is the similarity of the neighbor volumes between *N*_*i*_ and *N*_*j*_. Based on the constrution of 3D neighbor blocks, the NLM3D will restore noisy data from neighbor blocks within intraslices and interslices simultaneously which is helpful to preserve the structural information of different slices for MRI. Therefore, compared with classical NLM, the method has not only reduced the complexity of NLM significantly but also achieved superior denoising performance. The NLM3D has already applied ito super-revolution reconstruction of MRI [17] with different preselection step and computation formulation of *w*(*i*, *j*).

### 2.2. Multidimensional Principal Component Analysis

Although PCA has been applied in image denoising widely, most denoising algorithms based on PCA assume that data lie on vector space and usually process the vectorization operation to make image into a vector. The vectorization destroys the structural information about the neighborhood.

Instead of data in vector space, any multidimensional data can be considered as tensor data in multidimensional space. Each tensor data will be treated by tensor decomposition [18]. It is helpful to preserve the structural information and enhance the computational efficiency. At present, a large number of tensor algorithms have been presented and have a wide application in computer vision, pattern recognition, and machine learning [19].

Based on PCA on vector space, [20] has developed multidimensional principal component analysis (MPCA) for tensor data and has achieved outstanding performance. For MPCA, a high-dimensional image dataset can be expressed as a tensor dataset *X* = {*X*_1_,…, *X*_*M*_}, where *X*_*m*_ ∈ *ℝ* ^*I*_1_×⋯×*I*_*N*_^ is a *N* dimensional tensor and *M* is the number of samples in the dataset.

In tensor algebra, any tensor data *X*_*i*_ can be expressed based on Tucker decomposition model as follows [20]:

(4) Xm=Sm×U11×U22×⋯Un−1n−1×Un+1n+1×⋯×UNN,

where *U* ^(*n*)^ ∈ *ℝ* ^*I*_*n*_×*I*_*n*_^ is an orthogonal matrix. So we can get

(5) Sm=Xm×U11T×U22T×⋯Un−1n−1T×Un+1n+1T×⋯×UNNT.

The target of MPCA is to compute *N* orthogonal projective matrices {*U* ^(*n*)^ ∈ *ℝ* ^*I*_*n*_×*P*_*n*_^, *n* = 1,…, *N*} to maximize the total scatter tensor of the projected low-dimensional feature as follows:

(6) fUn,  n=1,…,N=arg maxUn⁡ Ψy=arg maxUn⁡∑m=1MYm−Y−2,

where $\overset{-}{Y}$ is the mean of tensor data *Y* and *Y*_*m*_ = *X*_*m*_ × _1_ *U* ^(1)^*T*^^ × _2_ *U* ^(2)^*T*^^ × ⋯_*n*−1_ *U* ^(*n* − 1)^*T*^^ × _*n*+1_ *U* ^(*n* + 1)^*T*^^ × ⋯×_*N*_ *U* ^(*N*)^*T*^^.

Due to the difficulty in the computation of *N* orthogonal projective matrices simultaneously, these *N* orthogonal projective matrices can be solved iteratively. Generally speaking, it is assumed that the projective matrices {*U* ^(1)^,…, *U* ^(*n* − 1)^, *U* ^(*n* + 1)^,…, *U* ^(*N*)^} are known; then we can solve the following optimized problem to obtain *U* ^(*n*)^:

(7) $$\begin{array}{c} \text{arg max}\overset{M}{\underset{m=1}{\sum }}\left(\;C_{m}^{\left(n\right)}C_{m}^{{\left(n\right)}^{T}}\;\right), \end{array}$$

where Cm=Xm-X-×U11T×U22T×⋯Un-1n-1T×Un+1n+1T×⋯×UNNT and $\overset{-}{X}$ is the mean of tensor data *X*. *C*_*m*_^(*n*)^ is the mode-*n* unfolding matrix of tensor *C*_*m*_. The paper [19] has proved the vector-based and 2DPCA can be considered as the special cases of MPCA.

### 2.3. Proposed Method

To the best of our knowledge, this is the first attempt to introduce MPCA to MRI image denoising. It should be noticed that image denoising based on MPCA is different from its application in machine learning.

For MRI denoising, 3D MRI is a 3rd-order tensor *X* ∈ *ℝ* ^*I*_1_×*I*_2_×*I*_3_^, *I*_1_ and *I*_2_ are the height and width of each MRI slice, respectively, and *I*_3_ is the number of slices, so 3D MRI can be considered as an image set *X* = {*X*_1_,…, *X*_*I*_3__}. The principal components can be computed by MPCA:

(8) Yi=Xi×U11T×U22T,

where *U* ^(*n*)^ ∈ *ℝ* ^*I*_*n*_×*I*_*n*_^, *n* = 1,2. Generally speaking, the first *K* principal components conserve most information of images. It is desirable to abandon smaller principal components to remove noise. There are various approaches to determine the value of *K*. The paper specifies a constant *K* to represent the number of the largest principal components corresponding to the largest *K* eigenvalues. After that, the restored image is expressed:

(9) X−i=Xi×U−11TU−11×U−22TU−22,

where ${\overset{-}{U}}^{\left(n\right)}\in {\mathbb{R}}^{K\times I_{n}}$, *n* = 1,2, and ${\overset{-}{X}}_{i}$ is the restored image. Consequently, the proposed algorithm can be summarized.

1. NLM 3D filtering is applied to denoise and obtain the initial 3D images.
2. Then the initial 3D images are processed by MPCA to remove noise furtherly.

## 3. Experiments

Several experiments were conducted to compare the proposed methods with related state-of-the-art methods.

In order to illustrate the performance of the proposed method, several experiments were conducted to compare the proposed methods with related state-of-the-art methods, including the 3D version of anisotropic diffusion filtering (3D-ADF) [3, 5], NLM3D [12], and OMNLM_LAPCA [16] on synthetic data and real clinical data. All experiments are performed on MATLAB R2015a.

There are some free parameters that need to be set to obtain optimal performance. For 3D anisotropic diffusion filtering, the integration constant is the maximum value, the number of iterations is 4, and the gradient modulus threshold is 70. For NLM3D and OMNLM_LAPCA, the radius of the search area is 5 and the radius of similarity area is 2. For the proposed method, the number of preserved largest principal components is 140 (see below).

Three kinds of quality measurement are used to evaluate the denoising performance. The first one is the signal-to-noise ratio (SNR), the second one is the peak signal-to-noise ratio (PSNR), and the last one is the structural similarity index (SSIM) [21].

The SNR is computed as follows:

(10) $$\begin{array}{c} \text{SNR}\left(\;x,\tilde{x}\;\right)=10\log_{10}\frac{\sum_{i,j}{\left(x\left(i,j\right)-\overset{-}{x}\right)}^{2}}{\sum_{i,j}{\left(x\left(i,j\right)-\tilde{x}\left(i,j\right)\right)}^{2}}, \end{array}$$

where *x* is the original image, $\overset{-}{x}$ is the mean of image *x*, and $\tilde{x}$ is the denoised image.

The PSNR is based on the root mean square error (RMSE) between the denoised image and original image:

(11) $$\begin{array}{c} \text{PSNR}=20\log_{10}\frac{255}{\text{RMSE}}. \end{array}$$

The SSIM is defined as follows:

(12) $$\begin{array}{c} \text{SSIM}\left(\;x,y\;\right)=\frac{\left(2\mu_{x}\mu_{y}+c_{1}\right)\left(2\sigma_{xy}+c_{2}\right)}{\left(\mu_{x}^{2}+\mu_{y}^{2}+c_{1}\right)\left(\sigma_{x}^{2}+\sigma_{y}^{2}+c_{2}\right)}, \end{array}$$

where *c*_1_ = (*k*_1_ *L*)^2^, *c*_2_ = (*k*_2_ *L*)^2^, *L* is the dynamic range, *k*_1_ = 0.01, and *k*_2_ = 0.03; *μ*_*x*_ and *μ*_*y*_ are the mean of images *x* and *y*, respectively; *σ*_*x*_ and *σ*_*y*_ are the standard noise variance of images *x* and *y*, respectively; *σ*_*xy*_ is the covariance of *x* and *y*.

### 3.1. Synthetic Data

**Figure 1:**
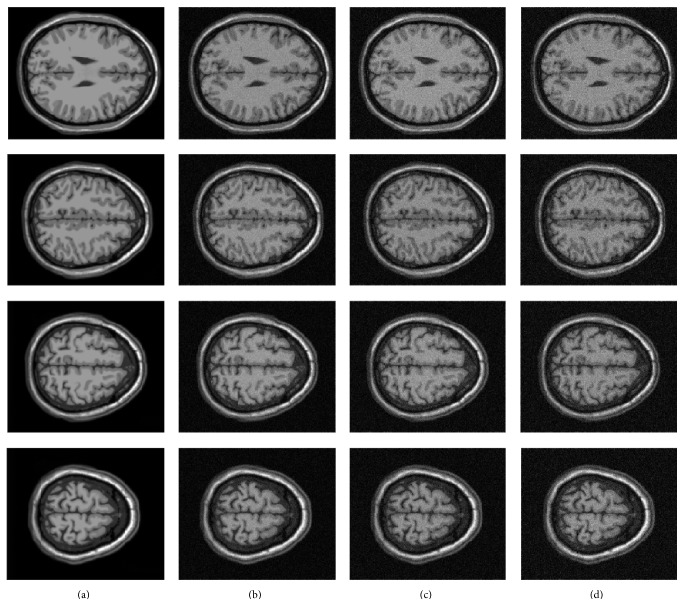
Example images of the BrainWeb database. (a) Noisy-free T1-w image, (b) noisy image corrupted with a Rician noise at 3%, (c) noisy image corrupted with a Rician noise at 4%, and (d) noisy image corrupted with a Rician noise at 5%.

In this part, the 3D T1-weighted MRI image in the well-known BrainWeb [22] dataset is used to evaluate the performance of the proposed method. The size of the dataset is 181 × 271 × 181 with 1 mm^3^ voxel resolution. To simulate Rician noise, zero mean Gaussian noise with 3–5% standard deviation is added to the real and imaginary parts of the 3D MRI images, as shown in Figure 1.

**Figure 2:**
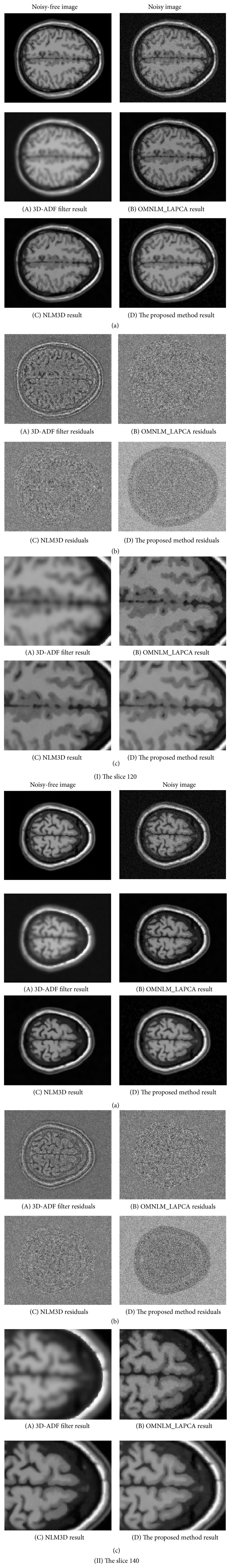
The noise-free image, noisy image, denoised images, residuals, and detail denoised images of different methods with 5% Rician noise for different slices.

The denoising performance of different methods with different noise levels is compared based on SNR, PSNR, and SSIM, as shown in Table 1. It is obvious that the proposed method is superior to the other three methods under the three evaluation measurements. The denoising images and corresponding residuals are shown in Figure 2. It is consistent with the measurement that the proposed method has the best visual effect.

**Figure 3:**
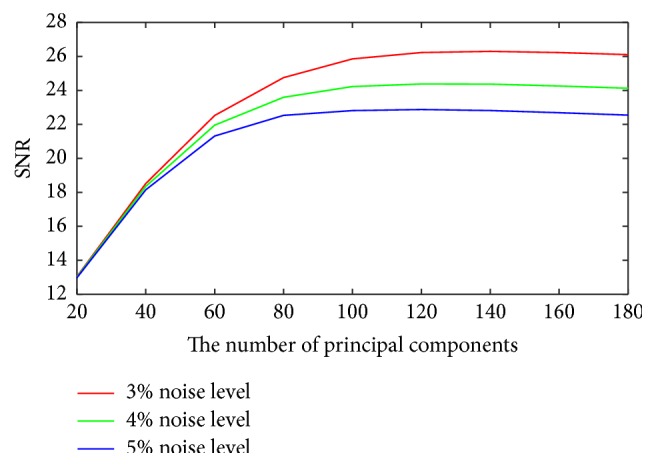
Plots of SNR versus the number of principal components for different noise levels.

**Figure 4 fig4:**
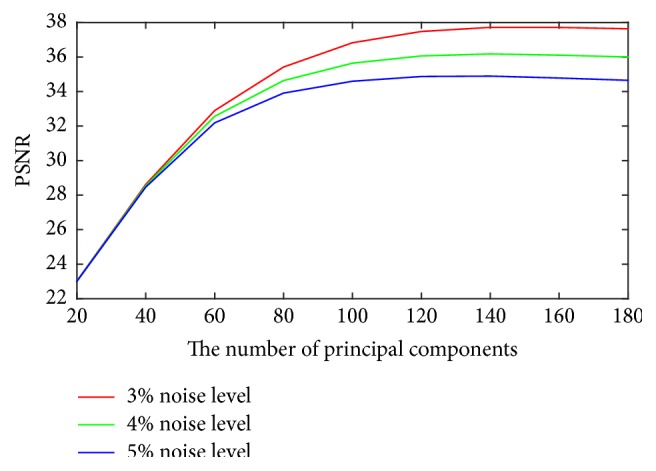
Plots of PSNR versus the number of principal components for different noise levels.

**Figure 5:**
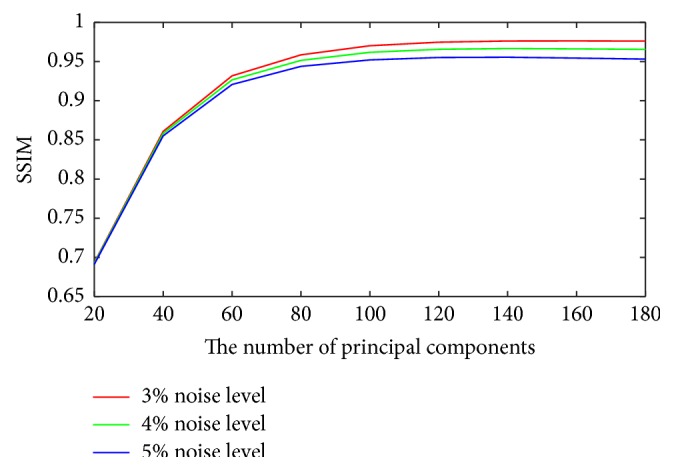
Plots of SSIM versus the number of principal components for different noise levels.

For the proposed method, it is required to determine the optimal number of the largest principal components. To study its influence on the denoising performance, the SNR, PSNR, and SSIM with different principal components with different noise levels are shown in Figures 3 [Fig fig4]–5. It can be seen that the optimal number is between 140 and 160 when the better measurements are obtained. So it is unreasonable to set a constant to choose the principal components. However, it is still an open problem in machine learning.

**Figure 6:**
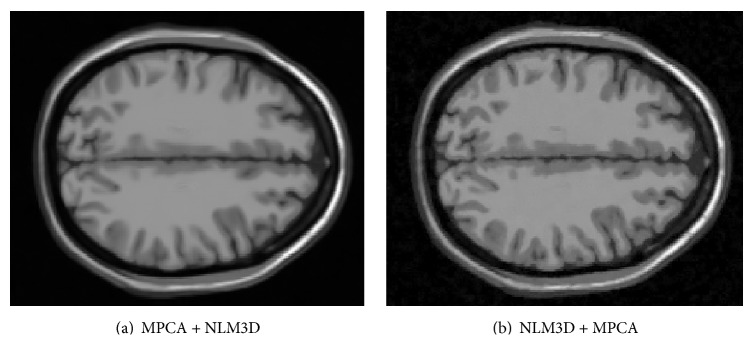
The denoised results with different order of MPCA and NLM3D (5% Rician noise). (a) First apply MPCA and then apply NLM3D but in (b) first apply NLM3D and then apply MPCA.

Based on the idea proposed in [16], the paper has presented a two-step MRI denoising algorithm, which employed NLM3D to restore 3D MRI followed by MPCA. To validate the proposed two-step method in the paper, we also have evaluated the order of MPCA and NLM3D.  Figure 6 has shown the denoised result with different order of MPCA and NLM3D. It can be seen that if we apply MPCA and then apply NLM3D, the detail information is lost and the denoised image is blurred. In contrast, the proposed steps in the paper have the capability to preserve the detail. The possible reason is that both of noise and detail are high frequency signals; the threshold technique of MPCA will remove the detail information while removing the noise if we conduct MPCA on the noisy image directly. Consequently, NLM3D is unable to restore the detail from the blurred neighboring block.

All denoising methods were performed in MATLAB 2015 on a Windows 7 computer equipped with an Intel Core i7-5600U, 2.6 GHz CPU and 8 GB RAM. To denoise typical 3D dataset with the size of 181*∗*217*∗*181, the corresponding computational time is listed in Table 2.

It cannot be denied that, compared with other algorithms, the proposed method will spend more time to denoise 3D MRI since it makes use of the 3D structure information from neighboring slices. It is also believed that the implementation of the proposed methods using MATLAB/C MEX techniques and parallel computations on graphic processing units may significantly further accelerate the filtering.

### 3.2. Validation on Real Clinical Data

**Figure 7:**
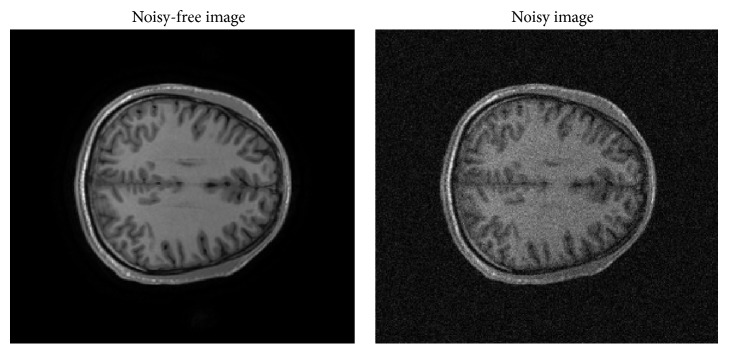
The example images of real clinical data and noisy data with 5% Rician noise.

To evaluate the proposed method on real clinical data, the experiments are conducted on real T1-w MRI data. The data were acquired on a GE MR750 3.0T scanner. The anatomical images were scanned using a T1-weighted axial sequence parallel to the anterior-commissure-posterior-commissure line. Each anatomical scan has 156 axial slices (spatial resolution = 1 mm × 1 mm × 1 mm, field of view = 256 mm × 256 mm, time repetition (TR) = 8.124 ms). The noisy-free image and noisy image are shown in Figure 7.

**Figure 8:**
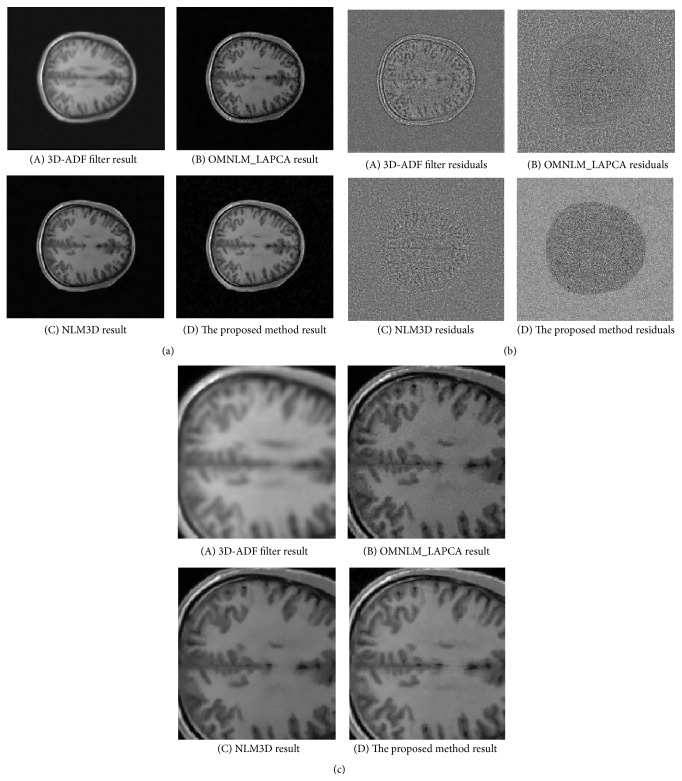
The denoised images, residuals, and detail denoised images of different methods with 5% Rician noise for different slices.

For real clinical data, the denoised results are shown in Figure 8 and Table 3. It can be seen that the proposed method also has achieved the best visual result. It may be that NLM3D with block representation restores noisy data from different neighboring slices. Moreover, in contrast with the principal components of PCA in vector space, MPCA seeks the principal components in tensor space, which has the capability to preserve the structure information of neighboring voxels and slices. At the same time, most image information focuses on the first *K* principal components, so the threshold technique is helpful to remove noise further.

## 4. Discussion

The paper has proposed a structure preserving MRI denoising algorithm. The method has integrated NLM3D and MPCA to restore noisy image from 3D neighborhood and has achieved a better result compared with some famous MRI denoising methods, such as 3D-ADF, OMNLM_LAPCA, and NLM3D. However, the confusing question of the proposed method is how to determine the optimal number of principal components, which will affect the denoising effect. So our next work will research the selection problem of principal components. We will consider the cumulative energy or the scoring of principal components in the future.

## Figures and Tables

**Table 1:** The comparison of denoising methods with different noise levels on T1 weighted MRI Images.

Denoising method	3% Rician noise			4% Rician noise			5% Rician noise		
	SNR	PSNR	SSIM	SNR	PSNR	SSIM	SNR	PSNR	SSIM
3D-ADF	13.7207	25.4512	0.7642	12.9584	25.4144	0.7624	12.1337	25.3515	0.7604
OMNLM_LAPCA	18.5248	35.0833	0.9495	16.0899	33.2808	0.9234	14.1684	31.7119	0.8927
NLM3D	25.9919	37.5705	0.976	24.0109	35.9133	0.965	20.8779	34.5518	0.9472
Proposed	**27.0346**	**38.5828**	**0.9864**	**25.0351**	**36.924**	**0.9755**	**22.9218**	**35.5598**	**0.9555**

**Table 2:** The comparison of computational time.

Denoising methods	The computational time(s)
3D-ADF	17.6905
OMNLM_LAPCA	32.8070
NLM3D	110.7295
Proposed	**111.0259**

**Table 3:** The comparison of different methods with 5% Rician noise.

Denoising method	SNR	PSNR	SSIM
3D-ADF	9.7543	24.8338	0.6717
OMNLM_LAPCA	9.7943	30.42	0.8415
NLM3D	18.7476	32.6647	0.9034
Proposed	19.7629	33.6679	0.9236
